# Tuberculosis prevalence and demographic characteristics of population in Azad Jammu and Kashmir (Pakistan): A retrospective study

**DOI:** 10.1097/MD.0000000000037787

**Published:** 2024-04-12

**Authors:** Soffia Khursheed, Samia Wazir, Muhammad Khurram Saleem, Ayesha Isani Majeed, Mumtaz Ahmad, Qudsia Umaira Khan, Arzu Jadoon, Amna Akbar, Sarosh Khan Jadoon, Sabahat Tasneem, Humayun Saleem, Mohammad Saleem Khan, Sarosh Alvi

**Affiliations:** a Pakistan Institute of Medical Science, Islamabad, Pakistan; b University Hospital, Bristol and Weston NHS Foundation Trust, Royal College of Physicians and Surgeons of Glasgow, Glasgow, UK; c Abbas Institute of Medical Sciences, Muzaffarabad, AJK, Pakistan; d CMH Lahore Medical College, Lahore, Pakistan; e Ziauddin University Hospital Karachi, Karachi, Pakistan; f CHPE Health Services Academy, Islamabad, Pakistan; g Resident Surgery, Combined Military Hospital, Muzaffarabad, Pakistan; h Health Services Academy, Islamabad, Pakistan; i MSPH Health Services Academy, Islamabad, Pakistan; j Chief Consultant Physician/Head of Department of Medicine DHQ Teaching, Hospital Kotli AJK, Kotli, Pakistan; k Teaching Faculty, University of Khartoum, Khartoum, Sudan.

**Keywords:** demography, geography, incidence, prevalence, serology, tuberculosis

## Abstract

Tuberculosis (TB) remains a serious problem for public health and a leading cause of death after COVID-19 and superior to even HIV/AIDS. It is a social health issue and can cause stigma and economic loss as the person cannot perform professionally due to lethargy caused by disease. It is a retrospective study done on data from National TB program Muzaffarabad chapter. The details were noted on SPSS and analysis was done to find important demographic characteristics. The total number of patients was 3441; among which 48.76% were males. Most of them (81.11%) belonged to the Muzaffarabad division of Azad Jammu and Kahmir (AJK). The microbiologically or culture positive cases were 440. Rifampicin resistance was present in 147 cases, further categorized as high (n = 143), very high (n = 3), or true positive (n = 1) resistance. Muti drug resistance was found in 19 cases. The microscopy culture is more sensitive (AUC = 0.511) than MTB/RIF or serology (AUC = 0.502) according to ROC. The rate of positive smear results is not very satisfactory in the present study as it cannot detect dormant or latent cases. There is a need to establish more sensitive tests for detection of cases and more research to combat the disease.

**Highlights**

○ Tuberculosis (TB) burden overwhelms the healthcare systems in most part of the world and Pakistan is one of the countries with highest burden.
○ Men are more affected than women
○ Cold weather advantages the mycobacterium tuberculosis and Azad Jammu and Kashmir (AJK) is an area that may favor its transmission due to weather
○ Age is an important factor in catching a TB infection and developing resistance.

## 1. Introduction

Tuberculosis (TB) is a communicable disease caused by acid-fast gram-positive bacillus bacteria belonging to genus Mycobacterium; *Mycobacterium tuberculosis* (MTB) being the most common. The other organisms are *M avium, M africanum*, and *M bovis*. MTB spreads through droplets produced by the infected individual’s cough. Meat from an infected animal or milk or milk products can spread *M bovis* to humans causing bovine tuberculosis that accounts for 10% of human TB cases.^[1]^ To survive and thrive within the host, the bacillus MTB has developed a wide range of survival mechanisms.^[2]^ The mycobacterium species are rare with morphological and biochemical differences within the lineage. It is likely that insertion and deletion led to the evolution of mycobacterium species with variable pathogenicity from a common ancestor. These species adapt to their host’s immune response and employee a various strategies evading immune attack to survive inside human body.^[3]^ In case of pulmonary tuberculosis (PTB), MTB enters the respiratory system, challenges host defense, develops its survival niche and releases an active form to infect another healthy host.^[4]^

Owing to the adaptive mechanisms and unique host-cell interaction, the pathogen is hard to detect, and it is impossible to track all transmissions till date. Thus, MTB had maintained its deadliness and it consumed over a billion individuals over 200 years.^[5]^ MTB is typically transmitted within families or neighborhood unevenly. Ongoing transmission, the co-location of risk factors and population characteristics determine the TB clustering.^[6]^ The economic burden and mortality associated with TB is substantial. Early diagnosis and detection of MTB has been a challenge as there is no gold standard for diagnosis. The main methods of sputum AFB (acid-fast bacilli), sputum genetic tests and culture, and chest x-ray are used to diagnose TB.^[7]^ Serology tests are now used to detect individuals’ exposure to specific pathogens and the immune response through antibodies. Serology testing can supplement and strengthen the diagnosis and detection of cases and help in surveillance for infectious diseases. So, it can help the public health systems if serology is included as a part of regular investigation for TB.^[8]^ Better diagnostic accuracy is desperately needed as the existing TB diagnostic methods have drawbacks. Various antibody isotypes and MTB antigens have been combined to enhance the diagnostic performance of classic serology; some of these combinations have demonstrated promising results for identification of active TB.^[9]^ With the use of fusion proteins (MPB70, MPB83, ESAT6, and CFP10) which are commonly present in the case of MTB, *M bovis* and *M caprae* exposure, an antigen coated MMEC; indirect ELISA was created for universal use. Fusion proteins A and G labeled with horseradish peroxidase (HRP) serve as secondary antibodies. As a result, numerous host-MTB complexes may be detected with serology.^[10]^

It is important to understand the epidemiology and local burden of disease to prioritize interventions and guide the formation of a good public health policy. The surveillance system for infectious diseases depends on the clinically reported cases, then determining the etiology and disease burden. It is then extended to normal population.^[8]^

Pakistan carries the highest burden (70% incidence) of tuberculosis in its region according to report published by WHO in 2022 followed by Afghanistan (9%), Iraq and Iran (1.3% each).^[11]^

## 2. Methodology

The study was conducted retrospectively, and the data was obtained from the National TB program in Azad Jammu and Kashmir (AJK), Pakistan, Muzaffarabad division. The objective of the study was to determine the prevalence and demographic characteristics of population in AJK. The data we could obtain after ethical approval from the ethical committee of AIMS hospital (ERB no. 9691) where the TB program center is located, spanned between January 2022 and December 2023.

The data for all those patients was included for which the data entries were complete, the specimen quantity for test was equal to or more than 5ml (because that is minimum amount in which an accurate test can be expected). The incomplete data entries were excluded.

The demographic characteristics of the patients were noted which included gender and age. The areas where the patients came from, and the home districts were also noted. The center for TB in Muzaffarabad mainly attends and receives patients from Muzaffarabad division of AJK but some patients from other areas like Abbottabad and Mansehra are also included as these individuals have a temporary residence in Muzaffarabad. The age of the patients was divided into categories each with an interval of 4 years like 1 year to 4 years. Categories were made up to 100 years. This was done according to WHO scale for age categories. The specimen was taken from pulmonary or extra pulmonary site. The color and appearance were different for each specimen type ranged from off white to yellow. Most of the specimens were mucopurulent and purulent. The type of specimen was also noted. The specimens were sputum, gastric fluid, and lavage, bronchial and bronchioalveolar lavage, pleural fluid, pus, and synovial fluid. All the details for each specimen were noted.

Next was the detail of how the patient presented at the TB center. It was noteworthy if the patient was a new case, already diagnosed CAT-I case or a CAT-II case with previous history of treatment, incomplete treatment, or relapse. The history of previous treatment for each patient was written. The details for the treatment response were taken like the type of treatment regimen being given (first line or second line treatment), the compliance to treatment was noted if the patients completed the treatment or not. The frequency of non-responsive cases was also recorded. The nonresponsive cases were then searched for their drug sensitivity tests. The drug sensitivity test was categorized as Monodrug resistant, polydrug resistant and multi-drug resistance. The patients were categorized into CAT-I and CAT-II patients, the second criteria were the history of treatment, and the third criteria was the completion of treatment. Hence, the patient’s categories were built and noted down. Rifampicin resistance was determined through a sputum culture test and further categorized as high, very high, or true positive based on the level of resistance. The frequency of each type of resistance was recorded.

After noting down all the details on an excel sheet, the data was transferred to an SPSS 25.0. The frequencies and percentages were calculated. Mean plots for age category were made. One way ANOVA test was performed to see the significance of age means with the classification at the time of the presentation at the TB center. The association of different factors with the resistance and smear positive result were determined and only the significant results were tabulated. The receiver operating curve (ROC) was constructed to find the specificity of 3 diagnostic criteria namely culture, Xpert MTB/RIF, and serology. The value of *P* being <.05 was considered as significant.

## 3. Results

**Figure 1.**
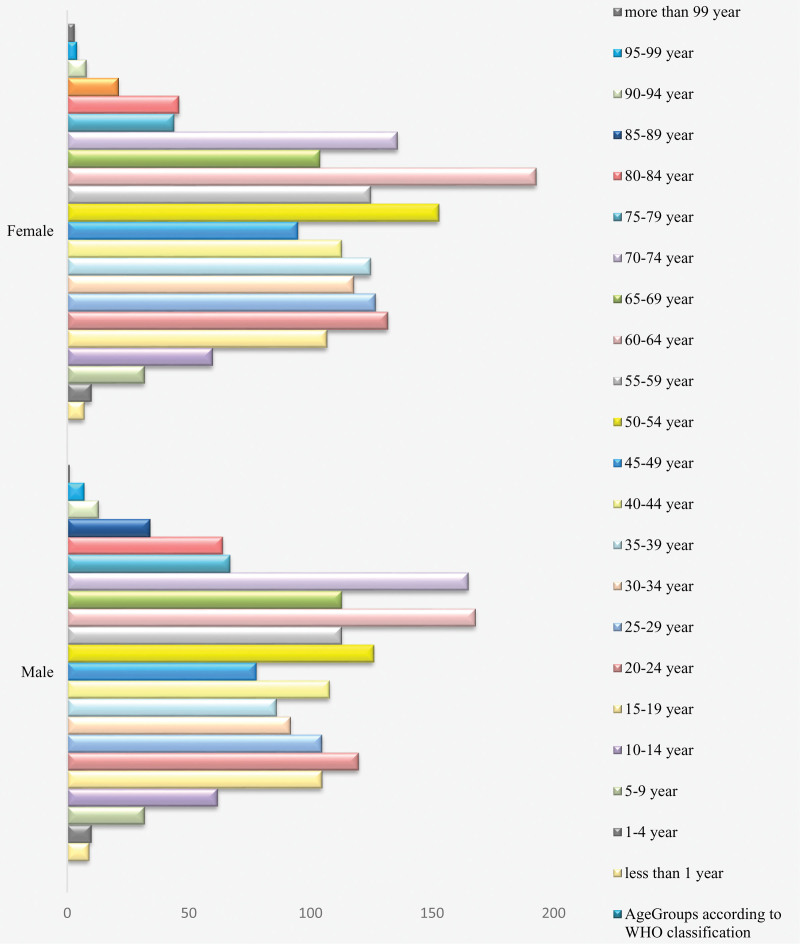
Age distribution (age groups according to WHO classification)* over gender. (*South East Asia RHO. Age Group Codelist [Internet]. apps.who.int. Available from: https://apps.who.int/gho/data/node.searo-metadata.AGEGROUP?lang=en).

Data for this study was obtained and analyzed retrospectively. The total number of patients was 3441; among which 48.76% were males. Most of them (81.11%) belonged to Muzaffarabad division of Azad Jammu and Kahmir (AJK). The patients who were observed in this cohort ranged in age from 2 weeks to 100 years. The highest frequency was observed between 60 to 64 years (n = 361), followed by 70 to 74 years and 50 to 54 years with 361 and 279 patients respectively. The details are given in Table 1. The age distribution over gender is given in Figure 1. The age range 60 to 64 years comprised of 168 males and 193 females scoring the highest frequency among the data set.

**Table 1:** Demographic characteristics of the population.

Variables		Frequency (Percent)
Gender	Male	1678 (48.765)
	Female	1763 (51.235)
Home District	Abbottabad	8 (0.232)
	Bagh	106 (3.080)
	Jhelum	227 (6.597)
	Kotli	2 (0.058)
	Mansehra	44 (1.279)
	Muzaffarabad	2791 (81.11)
	Neelum	244 (7.090)
	Poonch	19 (0.550)
Age groups according to WHO classification*	<1 year	16 (0.465)
	1–4 years	20 (0.581)
	5–9 years	64 (1.860)
	10–14 years	122 (3.545)
	15–19 years	212 (6.161)
	20–24 years	252 (7.323)
	25–29 years	232 (6.742)
	30–34 years	210 (6.103)
	35–39 years	211 (6.132)
	40–44 years	221 (6.423)
	45–49 years	173 (5.028)
	50–54 years	279 (8.108)
	55–59 years	238 (6.917)
	60–64 years	361 (10.491)
	65–69 years	217 (6.306)
	70–74 years	301 (8.747)
	75–79 years	111 (3.226)
	80–84 years	110 (3.197)
	85–89 years	55 (1.598)
	90–94 years	21 (0.610)
	95–99 years	11 (0.320)
	More than 99 years	4 (0.116)

* South East Asia RHO. Age Group Code list [Internet]. apps.who.int. Available from: https://apps.who.int/gho/data/node.searo-metadata.AGEGROUP?lang=en.

The specimen for the test was obtained from either pulmonary (n = 3344) or extra pulmonary (n = 97) site. The most common type of specimen was sputum (n = 3306) followed by a few samples of pleural fluid (n = 55) and gastric aspirate (n = 34). Frequency of samples from other sites was very rare. The quantity of specimen ranged between 5 mL to 20 mL; most of them quantified 5 mL (n = 1757). The appearance of the specimen was mucopurulent and purulent in most cases with other colors ranging from off white to yellow. The details are given in Table 2.

**Table 2:** The type, site, quantity, and appearance of the specimen obtained for diagnostic test.

Variables		Frequency (Percent)
Disease site	Pulmonary	3344 (97.181)
	Extra Pulmonary	97 (2.819)
Specimen type	Ascitic fluid	4 (0.116)
	Bronchial	2 (0.058)
	Bronchoalveolar	2 (0.058)
	Cerebrospinal Fluid	16 (0.465)
	Gastric Aspirate	34 (0.988)
	Gastric Lavage	4 (0.116)
	Gastric Fluid	2 (0.058)
	Pleural Fluid	55 (1.598)
	Pus Aspirate	13 (0.378)
	Spinal Epidural	2 (0.058)
	Synovial Fluid	1 (0.029)
	Sputum	3306 (96.077)
Quantity of specimen	5 mL	1757 (51.061)
	6 mL	492 (14.298)
	7 mL	87 (2.528)
	8 mL	817 (23.743)
	9 mL	73 (2.121)
	10 mL	211 (6.132)
	12 mL	1 (0.029)
	20 mL	3 (0.087)
Appearance	Brownish	4 (0.116)
	Cloudy white	33 (0.959)
	Colorless	4 (0.116)
	Greyish	5 (0.145)
	Mucopurulent	3248 (94.391)
	Off white	34 (0.988)
	Purulent	109 (3.168)
	Straw color	3 (0.087)
	Yellow	1 (0.029)

There were 2881 new patients who came for a tuberculosis diagnostic test with no previous history of TB diagnosis while 542 (15.75%) were those patients who already were diagnosed with TB but did not get any treatment. Patients who were already treated were CAT-II patients (n = 18); among them 2 were resistant to treatment and 10 patients had completed their treatment. Among the patients who were tested and were not new cases; 191 patients were prescribed first line treatment, 6 got second line treatment (CAT-II). Previous treatment was continued in 20 patients and the history of completed treatment was reported in 284 patients. Non-responsiveness to treatment was seen in 59 patients. Details are given in Table 3.

**Table 3:** Patient category at the presentation and according to treatment, and outcomes.

Variables		Frequency (Percent)
Category at presentation	CAT-I	542 (15.751)
	CAT-II	18 (0.523)
	New Case	2881 (83.726)
Previous treatment	No	3014 (87.591)
	Yes	427 (12.409)
Treatment at presentation	First line treatment	542 (15.751)
	Second line treatment	18 (0.523)
	New case	2881 (83.726)
Treatment outcome	First line treatment prescribed	191 (5.551)
	Second line treatment prescribed	6 (0.174)
	New case	2881 (83.726)
	Previous treatment continued	20 (0.581)
	Previous treatment completed	284 (8.253)
	Non-responsiveness to previous treatment	59 (1.715)
Patient category according to treatment and outcomes	CAT-I	50 (1.453)
	CAT-I-CT	4 (0.116)
	CAT-I-TC	59 (1.715)
	CAT-I-TNR	19 (0.552)
	PT-CAT-I	141 (4.098)
	PT-CAT-I-CT	15 (0.436)
	PT-CAT-I-TC	215 (6.248)
	PT-CAT-I-TNR	39 (1.133)
	PT-CAT-II	6 (0.174)
	PT-CAT-II-TC	10 (0.291)
	PT-CAT-II-TNR	2 (0.058)
	New case	2881 (83.726)

CAT-I = patients who have never received treatment, CAT-I-CT = Continued treatment for CAT-I, CAT-II = history of previous treatment-relapse or failure, CAT-I-TC = Treatment completed for CAT-I, CAT-I-TNR = Treatment not responsive for CAT-I, PT = previous treatment, PT-CAT-II = previously treated for CAT-II, PT-CAT-II-TC = previously treated for CAT-II treatment completed, PT-CAT-II-TNR = previously treated for CAT-II and treatment was non-responsive.

Table 4 represents the results of the results of the laboratory tests. The microbiologically or culture positive cases were 440 (Table 4). The rifampicin resistance was present in 147 cases. It can be further categorized as high (n = 143), very high (n = 3) and true positive (n = 1). Muti drug resistance was found in 19 cases.

**Table 4:** Results of laboratory investigations.

Variables		Frequency (Percent)
Culture	Negative	3001 (87.213)
	Positive	440 (12.787)
Xpert MTB/RIF Assay	Error/invalid	161 (4.679)
	Not detected	2760 (80.209)
	Traces	86 (2.499)
	Very low	63 (1.831)
	Low	146 (4.243)
	Low-medium	3 (0.087)
	Medium	75 (2.180)
	High	143 (4.156)
	High RrD	3 (0.087)
	True positive	1 (0.029)
Serology	Error	129 (3.749)
	No trace	3304 (96.019)
	Low trace	1 (0.029)
	Intermediate trace	7 (0.2030)
Resistance	No resistance	3372 (97.995)
	Monodrug resistance	17 (0.494)
	Polydrug resistance	33 (0.959)
	Multidrug resistance	19 (0.552)
MDR_TB	No	3422 (99.448)
	Yes	19 (0.552)

High RrD = Rifampicin resistance detected, MDR_TB = multi drug resistance tuberculosis, MTB/RIF = mycobacterium tuberculosis/rifampicin resistance.

**Figure 2.**
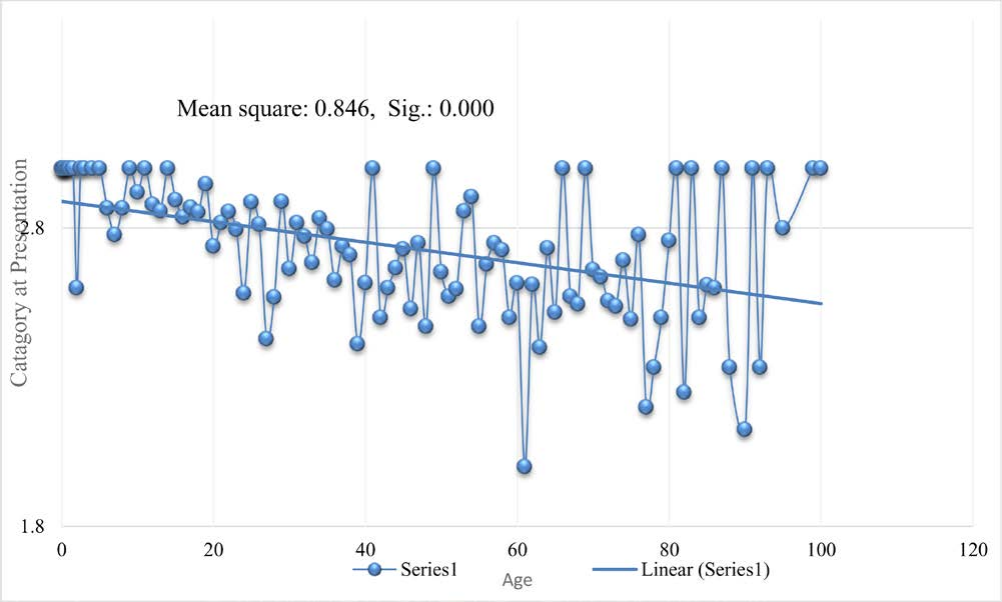
Means plots for age according to the category of patients presented at the hospital.

**Figure 3.**
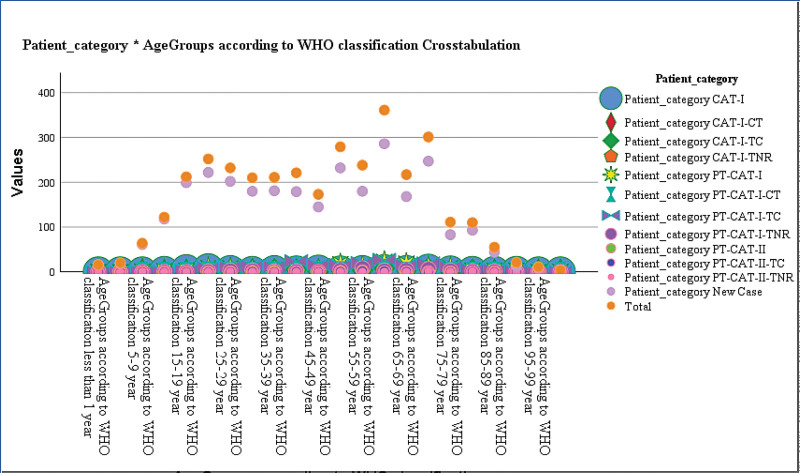
Distribution of patients ‘categories according to age.

The categories in which the patients were divided on basis of treatment and outcomes and the smear positive results were associated (*P* < .000), the details are given in Table 5. Out of total 440 patients who were tested positive, 358 were new cases. There were 215 patients who had completed CAT-I treatment (PT-CAT-I-TC); they were all smear negative. Similarly, age groups had a positive association with categories. As described earlier the highest frequency was observed between 60 to 64 years (n = 361), among which 286 were new cases, 22 were CAT-I and 27 were CAT-I who had completed treatment and were smear negative. Means plots for age and categories at presentation are given in Figure 2 and the distribution of age according to patients ’categories based on treatment and outcomes is given in Figure 3.

**Table 5:** Association of patients categories with age and culture positive tests.

Age groups according to WHO classification	CAT-I	CAT-I-CT	CAT-I-TC	CAT-I-TNR	PT-CAT-I	PT-CAT-I-CT	PT-CAT-I-TC	PT-CAT-I-TNR	PT-CAT-II	PT-CAT-II-TC	PT-CAT-II-TNR	New Case	*P* value
<1 year	0	0	0	0	0	0	0	0	0	0	0	16	.000
1–4 year	0	0	0	0	0	0	1	0	0	0	0	19	
5–9 year	0	1	0	0	1	0	1	0	0	0	0	61	
10–14 year	1	0	1	0	2	0	0	0	0	0	0	118	
15–19 year	5	0	1	0	3	0	4	0	0	0	0	199	
20–24 year	7	0	1	1	9	1	10	1	0	0	0	222	
25–29 year	3	1	3	0	8	0	12	3	0	0	0	202	
30–34 year	1	0	5	1	5	0	13	3	1	1	0	180	
35–39 year	3	0	6	1	5	1	12	1	1	0	0	181	
40–44 year	2	0	5	2	4	3	24	1	0	1	0	179	
45–49 year	4	0	1	0	6	1	16	0	0	0	0	145	
50–54 year	2	0	5	1	17	1	15	4	1	1	0	232	
55–59 year	3	0	4	3	12	4	20	8	2	2	0	180	
60–64 year	5	2	8	3	22	3	27	3	0	2	0	286	
65–69 year	3	0	4	3	18	0	16	4	0	1	0	168	
70–74 year	6	0	8	2	11	1	19	6	0	0	1	247	
75–79 year	2	0	1	1	9	0	12	3	0	0	0	83	
80–84 year	1	0	4	0	3	0	6	1	0	1	1	93	
85–89 year	1	0	0	1	3	0	5	1	0	0	0	44	
90–94 year	1	0	2	0	3	0	2	0	0	0	0	13	
95–99 year	0	0	0	0	0	0	0	0	1	1	0	9	
more than 99 years	0	0	0	0	0	0	0	0	0	0	0	4	
	50	4	59	19	141	15	215	39	6	10	2	2881	
Culture													
Negative	35	2	58	16	90	15	215	33	2	10	2	2523	.000
Positive	15	2	1	3	51	0	0	6	4	0	0	358	
	50	4	59	19	141	15	215	39	6	10	2	2881	

**Figure 4.**
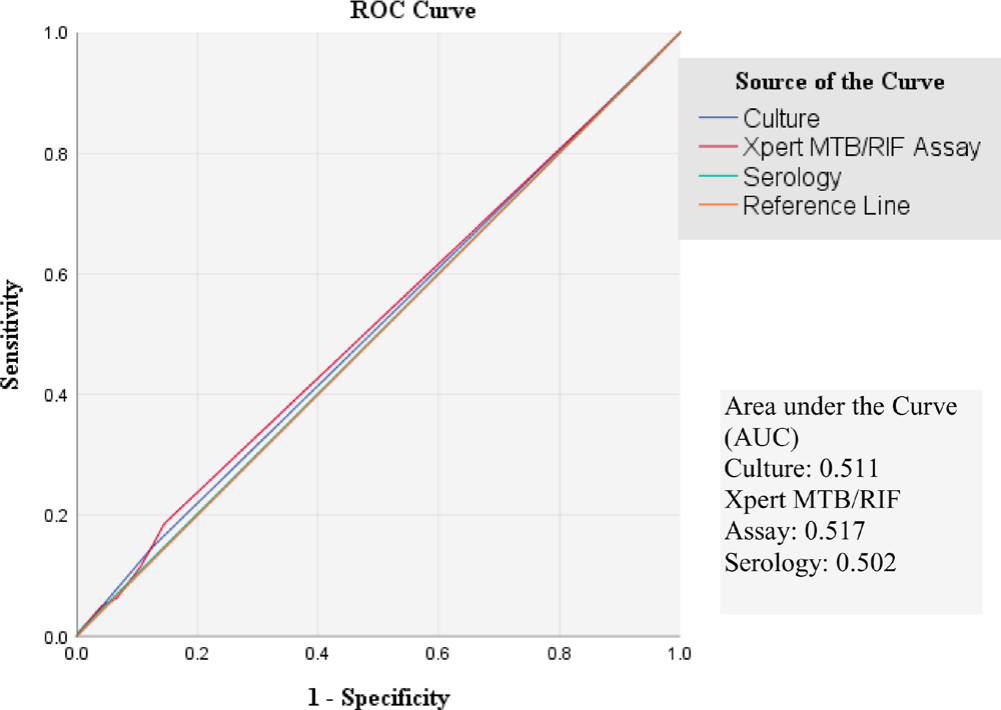
ROC curve built for diagnostic tests to detect positive cases of Tuberculosis.

**Figure 5.**
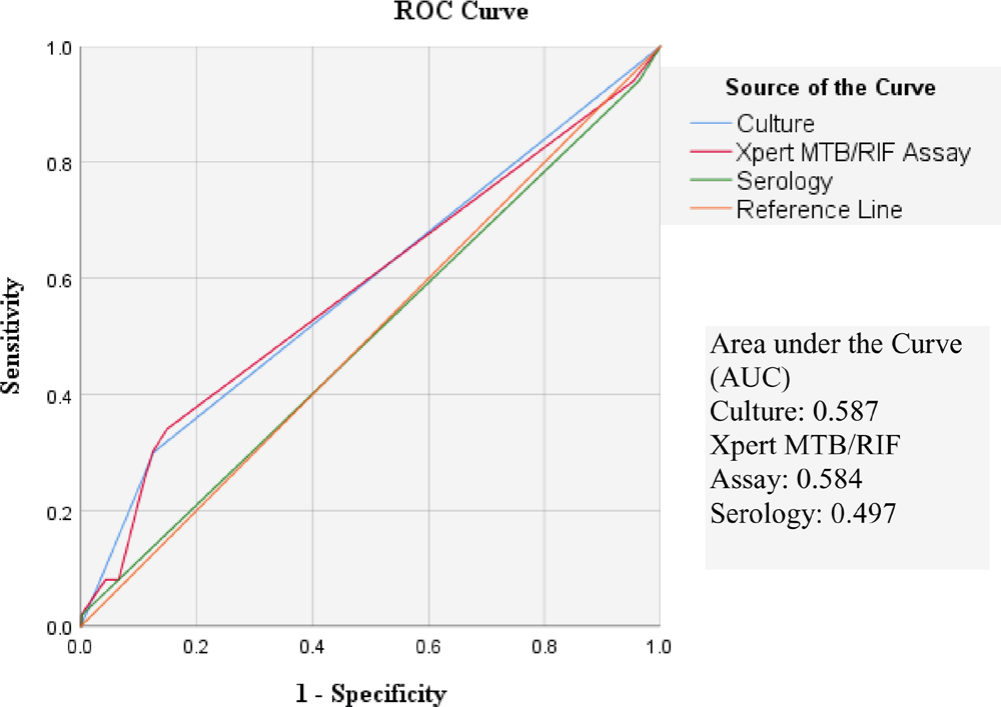
ROC curve built for diagnostic tests for drug resistance.

The receiver operating characteristics (ROC) curves are given in Figures 4 and 5 for smear positive results and resistance respectively. Microscopy culture is more sensitive than MTB/RIF or serology according to these curves.

## 4. Discussion

Tuberculosis (TB) is the granulomatous infection caused by mycobacterium tuberculosis (MTB) characterized by chronic cough and fever more than 2 weeks, weight and appetite loss, nocturnal sweats, chest pain and blood in sputum. TB is the second leading killer among infectious diseases after COVID-19 (exceeding HIV). In 2022, 10.6 million new cases of TB were reported; among them 5.8 million were men, 3.5 million women and 1.3 million children. TB mortality was 1.3 million in 2022 worldwide.^[12]^ Tuberculosis infection overwhelms the healthcare systems despite the availability of chemotherapy and neonatal vaccine because MTB preserves a significant metabolic versatility. The organism has modified and augmented the metabolic pathways of its ancestors to thrive within its obligatory human host.^[13]^

About 3 million people are thought to remain undiagnosed or misdiagnosed or untreated. Early detection of TB cases is difficult, especially in setting with limited sources and among marginalized populations.^[14]^ In the present study 3441 patients came for a test for confirmation of TB and only 440 were smear positive. The patients were mostly categorized according to clinical signs and symptoms.

The available diagnostic approaches for TB in humans are insufficient. For TB screening, early differentiation and monitoring treatment, host biomarkers are proposed as a promising diagnostic tool. “Multi omics” methodologies assist in host biomarker studies and provide comprehensive understanding of the host-TB interactions.^[15]^ In our country, the gene Xpert, Xpert MTB/RIF, drug sensitivity tests, microscopy culture and serological tests are provided by the National TB Program. The exact test for accurate results is not known.

System serology and flow cytometry are the 2 most recent techniques that can provide a high degree of accuracy in detecting MTB by measuring immunological markers unique to MTB. Second generation interferon-gamma release assays (IGRAs) might be used to segregate active TB cases especially in low incidence areas.^[16]^ Serology in our study had the least specificity for diagnosis (area under curve in ROC 0.502) and drug resistance (area under curve in ROC 0.497) which is very low.

Larger studies are needed to confirm characteristics related to TB. A cohort of 10,087,903 individuals was observed in Korea under the National Health Screening Program and the TB incidence was 0.92 per 1000-person years.^[17]^ There were 25 culture confirmed Tb cases reported among 9877 individuals in Dhaka. The new cases were 253 per 100,000 with only 1 child and 52% with a BMI <17 kg/m^2^. Twenty isolates yielded 13 spoligo type patterns, 5 clusters and 12 bacteria each.^[18]^ In our study 440 cases were marked positive by culture analysis.

The geographical area is important for TB transmission because humidity affects the process. Using the best weather conditions for the lowest risk of infection, the risk of contracting TB is greatest when it is cold, humid and rainy.^[19]^ The present study was conducted in Azad Jammu and Kashmir (AJK) which is a cold area as compared to other geographical areas in Pakistan.

The incidence and prevalence of TB varies greatly from one region of the world to another owing to specific characteristics of each region. From 2001 to 2019, 475 cases were reported in Italy which is a low incidence country. 67.6% were Italians and 32.4% were foreigners among which 75.3% were residents and others were irregular residents. The incidence was greater in foreigners (31.7) than the Italians (2.7) per 100,000 people. Italians averaged 48.4 years while resident foreigners were 32.7 years and irregular foreigners 19.6 years of age on average.^[20]^ In our study no foreigners were reported but there were some patients from outside the district like Mansehra and Abbottabad.

Tuberculosis in the world: The cumulative incidence of TB in Iran was 18.52 (23.84 in 2024 and 15.02 in 2019) per 100,000 between 2014 and 2019. Recovery rate (85.34%), completion of treatment course (2.05%), loss to follow-up (1.17%), treatment failure (4.4%) and death (6.45%) were all reported. Men and rural areas were more affected.^[21]^ Sabah state in Malaysia accounted for 20% of all reported TB cases between 2012 and 2018, even though Sabah makes up 10% of total population. The prevalence of TB was highest in older males. Just 4.6% of the incidents included children less than 15 years of age. A substantial percentage had moderate or advanced manifestations on chest x-ray (58% and 81% respectively) suggesting a high probability of late detection. Treatment success rate was 83% (81–85%) in the case of sensitive TB.^[22]^

In Brazil, the PTB was found in 81% of the patients, the rate of TB cases was 76.8% with mortality of 0.8%. In children under 5 years, helminth co-infection, malaria, vaccine related issues, persistent viral infections and hypovitaminosis D were risk factors. Tuberculin skin test was applied for risk identification.^[23]^ When age-standardized rates were observed, the prevalence of TB in relation to 45 to 59 years and less than 1 year age groups and males was highest in 2017. Age-standardized DALY rates fell in all states of Brazil between 1990 and 2017.^[24]^ The bacteriological data in Indonesia shows that there were 759 (589–960) cases per 100,000.^[7]^ A total of 2086 TB cases were reported in China out of which 1402 were extra pulmonary tuberculosis (EPTB) only. Age more than 2 years is an independent predictor and female gender was risk factors for EPTB, presence of a comorbid and severity of symptoms were crucial factors for overall TB.^[25]^ In a study in India, 75% of the TB cases were found in age group 18 to 59 years, 845 were from rural areas. EPTB was diagnosed in 52% and only 18% had a history of previous treatment. Microbiological confirmation was more common in PTB (89.6%) than in EPTB (17.3%). Additionally, rural areas had a higher prevalence of EPTB than PTB.^[26]^ Population in Inner Mongolia experienced increase in burden of disease between 2016 and 2018. The vulnerable population was elderly men and workmen, while female and children were less vulnerable.^[27]^ In our study the, although lesser number of male patients were tested than females, the number of male patients test positive is more than female.

Almost 10% of TB cases are found in young population,^[1]^ so, WHO recently has shifted attention to childhood TB. Children are more vulnerable and catch MTB more rapidly, develop severe symptoms and disseminated forms of disease and higher chances of active infection. Bacteriologically confirmed cases ranged from 45 to 799/1,000,000 in Asia-pacific area while in Africa it was 162 to 462/1000, 000. The adolescent population in endemic areas is at risk and should be given attention immediately.^[28]^ More than 1 million adolescents under 15 years contract TB infection annually (WHO estimates). In some areas 25% of the new cases are drug resistant. Major advancements have been made in the past 15 years in the epidemiological reporting of TB in children and adolescents in Spain. Some of them are the development of new immunological tests, the availability of molecular methods for rapid microbiological diagnosis and detection of drug resistant variants. In treating TB, the discovery of novel second line anti TB drugs, including those intended for pediatric use and the validation of short treatment courses for certain patients based on clinical trials are all new developments.^[29]^ In our study the most vulnerable population was the age of 60 to 64 followed by the age of 70 to 74 and then 50 to 54 years.

In the population at Thailand-Myanmar border, children were the most vulnerable population, 94% of them were diagnosed on basis of clinical criteria. Of the patients, 95% completed their treatment but 10.6% had unsatisfactory outcomes with 15 fatalities and one treatment failure. Bacteriologically confirmed TB, co-existing HIV and EPTB were independent risk factors.^[30]^ In Pakistan 55.3% of the 2176 individuals under the age of 14 years diagnosed with TB were female and 57.3% were from urban region. PTB was found in 76.9% cases, of which 13.3% were sputum smear positive. The treatment success rate was 92.4% while the failure was associated with sputum smear positive cases, rural inhabitants, and retreated patients. The highest risk for contracting PTB was linked to age ≤ 2 years, BMI less than normal and male gender.^[31]^ TB is an illness with social impact acting as a social welfare barometer. Poor and crowded living space, higher population growth, smoking, malnutrition, alcoholism, illiteracy, child marriages and ignorance of the cause and spread of TB are some of the societal factors that facilitate development and progression of TB. Another risk factor is diabetes which is present in 20.8% smear positive cases and 14.8% of TB overall.^[1]^ In present study children were not on more threat than elders.

Individuals who are at higher risk of developing TB are called vulnerable population; their socioeconomic status usually restricts them from assessing healthcare facilities. The comprehensive assessment of vulnerable population worldwide exhibited a pooled prevalence of 25% in homeless, prisoners, asylum seekers, refugees and HIV positive individuals compared to general population. There was little evidence specific to sex workers, homosexual men, transgender, nomads, and minors. The higher medians of TB in vulnerable population emphasize the need for implored integration of these groups, including focused offers for identification, screening, and preventive measures and treatment.^[32]^ Three million refugees were tested for TB and the incidence was 19 to 754 and 18.7 to 535 cases per 1,000,000 people, being most common in Asia and Africa. The prevalence of TB is higher in the migrant population than in the host country. There is a need to strengthen the TB prevention and control programs among refugees.^[33]^ Our study has not reported any HIV or any other serious comorbid condition.

Another vulnerable group is the population inflicted with latent tuberculosis infections (LTBI) that are usually asymptomatic. About 5% to 10% LTBI has a lifelong risk of developing active TB. The detection of LTBI and treatment of active cases is crucial. It will be extremely difficult to meet the End TB target of 2035 if the population of latently infected people cannot be found and treated.^[34]^ LTBI patients experienced worst mental health issues than the non-LTBI patients as the LTBI patients experience fear, stigma and dread.^[35]^ While many treatment options are available to cure TB, it is unclear how nations will meet 2035 targets of eliminating TB through national TB programs. Limitations are lack of an accurate and timely diagnosis for MTB infection and drug resistance.^[36]^ There was no test through which we could confirm the latent tuberculosis. The major portion of culture negative results indicate that there are chances that cases of Tb go undiagnosed.

Conclusion: The disease TB is one of the most dangerous diseases causing death. The rate of positive smear results is not very satisfactory in the present study as it cannot detect dormant or latent cases. There is a need to establish more sensitive tests for detection of cases and more research in order to combat the disease.

## Acknowledgments

Direct technical help in the form of data set by Abbas Institute of Medical Sciences. Furthermore, indirect assistance was provided by Google Scholar, PubMed, and Science Direct SciFinder® and Sci-hub in the form of literature.

## Author contributions

**Conceptualization:** Samia Wazir, Qudsia Umaira Khan, Mohammad Saleem Khan.

**Data curation:** Amna Akbar, Humayun Saleem.

**Formal analysis:** Amna Akbar, Sabahat Tasnem, Humayun Saleem.

**Methodology:** Samia Wazir, Arzu Jadoon.

**Resources:** Sarosh Khan Jadoon.

**Software:** Arzu Jadoon, Sarosh Alvi.

**Supervision:** Mumtaz Ahmed, Mohammad Saleem Khan.

**Writing – original draft:** Soffia Khursheed, Muhammad Khurram Saleem.

**Writing – review & editing:** Soffia Khursheed, Muhammad Khurram Saleem, Ayesha Isani Majeed, Qudsia Umaira Khan, Sarosh Khan Jadoon.
